# Identification and regulation of an alternative PTS for disaccharide utilization in *Clostridium acetobutylicum*

**DOI:** 10.1128/aem.00709-25

**Published:** 2025-10-08

**Authors:** Zhenxing Ren, Zili Qiu, Yali Tian, Mengcheng You, Chenggang Xu

**Affiliations:** 1 Institute of Applied Chemistry, Shanxi University 12441, Taiyuan, Shanxi Province, China; 2 College of Animal Science and Technology and College of Veterinary Medicine, Zhejiang A&F University, Key Laboratory of Applied Technology on Green-Eco-Healthy Animal Husbandry of Zhejiang Province, Zhejiang Provincial Engineering Research Center for Animal Health Diagnostics & Advanced Technology, Zhejiang International Science and Technology Cooperation Base for Veterinary Medicine and Health Management, China-Australia Joint Laboratory for Animal Health Big Data Analytics 722545, Hangzhou, Zhejiang, China; 3 Key Laboratory of Chemical Biology and Molecular Engineering of Ministry of Education, Institute of Biotechnology, Shanxi University 12441, Taiyuan, Shanxi Province, China; 4 Shanxi University of Chinese Medicine, Jinzhong, China; Danmarks Tekniske Universitet The Novo Nordisk Foundation Center for Biosustainability, Kgs. Lyngby, Denmark

**Keywords:** *Clostridium acetobutylicum*, disaccharide PTS, *bgl* operon, RAT

## Abstract

*Clostridium acetobutylicum* is an important solventogenic bacterium capable of acetone-butanol-ethanol fermentation by utilizing a variety of carbon sources. It employs the transport systems of the phosphoenolpyruvate:sugar phosphotransferase systems (PTSs) to assimilate various saccharides. Here, we investigated a β-glucoside PTS (*bglT*) encoded by the *bgl* operon (*bglGTH*) in *C. acetobutylicum*, which showed significant expression in response to cellobiose and sucrose. Interestingly, *bglT* is not essential for the transport of these sugars, as *C. acetobutylicum* possesses dedicated PTSs for the uptake of each individual sugar. We further elucidated the regulatory mechanism of *bglT*, which is governed by an upstream anti-transcriptional termination factor (*bglG*). A putative ribonucleic antiterminator (RAT) was identified upstream of *bglG* and *bglT*. Inactivation of *bglG* led to consistent read-through frequencies of the genes downstream of the RAT, irrespective of the sugar present. Conversely, complete removal of RAT elevated the transcriptional levels of downstream genes, while partial deletion of RAT, causing a long stem-loop structure (terminator), resulted in transcription termination. These findings provide novel insights into the regulatory mechanisms controlling sugar utilization in *C. acetobutylicum*.

**IMPORTANCE:**

Cellulose, the most abundant organic compound on Earth, is primarily found in plant cell walls and can be broken down into sugars such as cellobiose. These sugars are crucial for microbial fermentation, especially in biofuel production. *Clostridium acetobutylicum*, a promising microorganism for producing short-chain alcohol chemicals, can utilize cellulose degradation products as a carbon source for fermentation. This study identifies the transport systems involved in the utilization of cellobiose and other disaccharides in *C. acetobutylicum* and analyzes their regulatory mechanisms. Understanding these pathways is essential for enhancing biofuel production from plant biomass.

## INTRODUCTION

*Clostridium acetobutylicum* is a gram-positive, anaerobic, spore-forming industrial microorganism renowned for its ability to utilize a wide range of carbon sources for acetone-butanol-ethanol fermentation (1, 2). Among these solvents, butanol stands out as a promising next-generation biofuel due to its high energy density and low volatility, making it an excellent substitute for gasoline. Meanwhile, acetone and ethanol are valuable chemicals with broad applications in the chemical industry (3). Consequently, enhancing the carbon source utilization efficiency of *C. acetobutylicum* to boost solvent yield has become a focal point in bioenergy research (4).

The ability of *C. acetobutylicum* to efficiently metabolize various sugars is attributed to its sophisticated sugar transport and metabolism systems (5). Phosphoenolpyruvate:sugar phosphotransferase systems (PTSs) transport numerous monosaccharides, disaccharides, and sugar alcohols (6). The first PTS identified in *C. acetobutylicum* was specific to sucrose (7). Subsequent advances in genome sequencing have revealed the presence of 13 PTSs in the *C. acetobutylicum* genome (8). Among these, PTSs responsible for transporting glucose (9), cellobiose (10), sucrose (7), mannose (11), fructose (12), maltose (13), lactose, and galactose (14) have been characterized. Notably, cellobiose, a key intermediate in cellulose degradation, holds particular importance in the conversion of lignocellulosic biomass into liquid fuels and chemicals.

Servinsky et al. (5) first inferred from transcriptome surveys under varying carbon sources that *C. acetobutylicum* encodes two candidate cellobiose PTSs, encoded by CA_C0383–CA_C0386 and CA_C1407–CA_C1408. The CA_C0383–CA_C0386 locus has since been proven as a functional cellobiose transporter and shown to be stringently regulated by the CelR repressor in concert with sigma factor σ54 (SigL) (10). Nevertheless, the inactivation of *celR* does not completely block the utilization of cellobiose, confirming the existence of an alternative cellobiose transport system.

In this study, we conducted a comprehensive functional and regulatory analysis of another potential cellobiose PTS encoded by CA_C1407-CA_C1408, referred to as the *bgl* operon due to its role in encoding a β-glucoside-specific PTS. Through gene inactivation and transcriptional profiling, we elucidated the mechanisms underlying the efficient uptake and utilization of disaccharides, particularly cellobiose, which is crucial for enhancing the conversion of lignocellulosic biomass into biofuels.

## RESULTS

### The *bgl* gene cluster is potentially involved in metabolism of disaccharides

Although *C. acetobutylicum* possesses the *cip-cel* gene cluster, which encodes cellulosomes responsible for the degradation of lignocellulose, its ability to grow on cellulose is hindered by the low yield and activity of these cellulosomes. Nonetheless, *C. acetobutylicum* is capable of utilizing the breakdown products of cellulose, such as cellobiose. Building on the observations of Servinsky et al. (5), who noted that *C. acetobutylicum* harbors a second, cellobiose-inducible PTS gene cluster (CA_C1406–CA_C1408) in addition to the canonical CelABC system, we undertook to characterize this gene cluster in detail.

**Fig 1:**
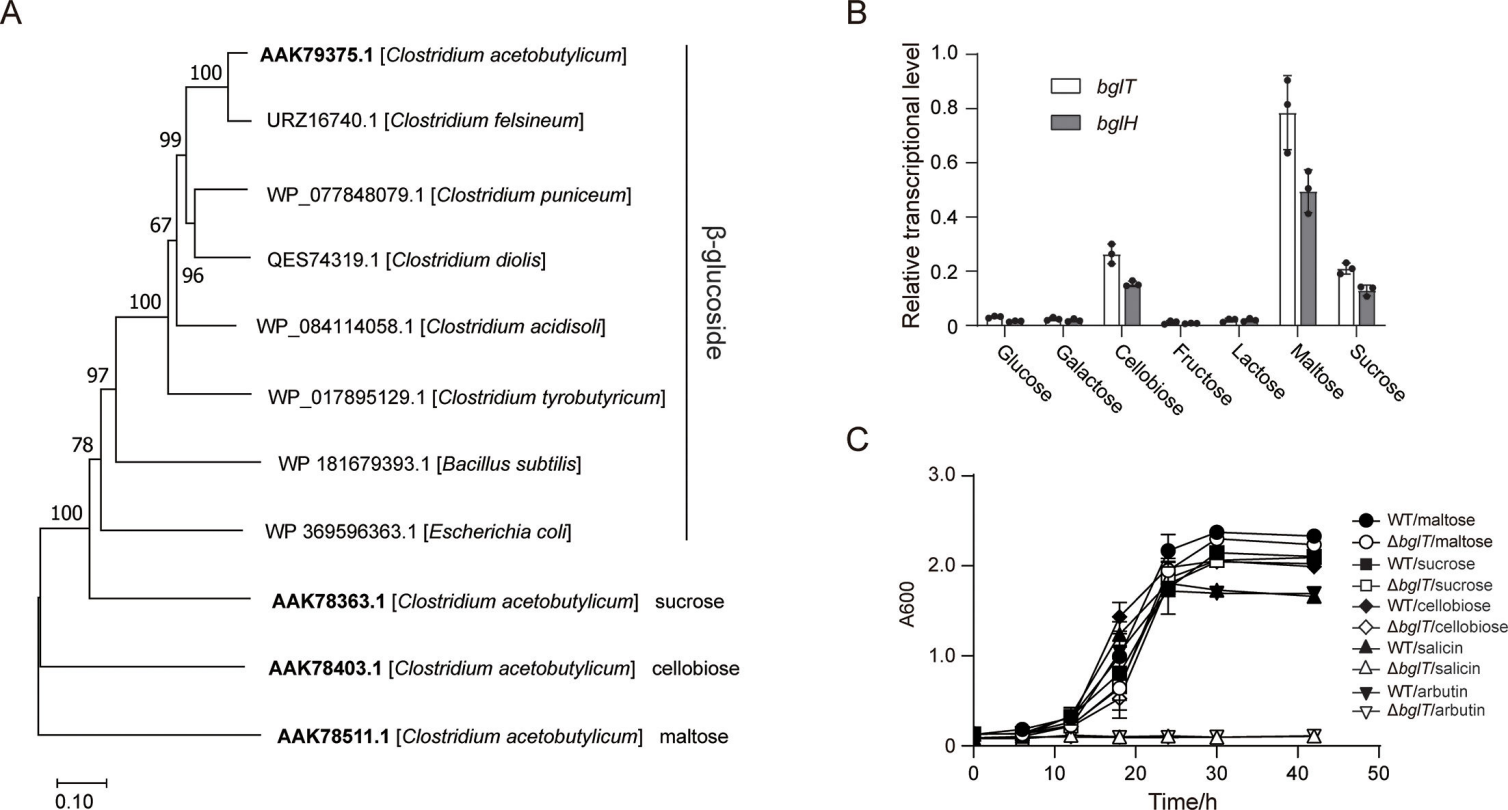
Identification of β-glucoside PTS encoded by the *bgl* operon in *C. acetobutylicum*. (**A**) Phylogenetic analysis of β-glucoside PTS components from solventogenic *Clostridium* species, *Escherichia coli*, *Bacillus subtilis*, and selected *C. acetobutylicum* carbohydrate PTSs. The tree was constructed using a maximum composite likelihood method with neighbor-joining algorithms and supported by 100 bootstrap replicates. The scale bar represents 0.10 amino acid substitutions per site. (**B**) Transcription levels of *bglT* and *bglH* under various carbon sources. Transcription levels were quantified by quantitative PCR (qPCR) using gene-specific primers and normalized to the 16S rRNA gene as an internal reference. Bars represent mean ± standard deviation (SD) (*n* = 3), and individual data points are shown. (**C**) Growth curves of *C. acetobutylicum* wild type and Δ*bglT* mutant grown on salicin, arbutin, maltose, sucrose, or cellobiose. Experiments were performed in triplicate, and bars indicate the standard deviation.

The gene cluster comprises a β-glucoside-specific PTS IIBCA component (CA_C1407, named *bglT*), a putative phospho-β-glucosidase (CA_C1408, *bglH*), and a BglG-type transcriptional regulator (CA_C1406, *bglG*). Expression of this three-gene unit is controlled by the BglG protein acting on RAT elements. Notably, the conserved gene order *bglG-bglT-bglH* is maintained across solventogenic *Clostridium* species. To investigate evolutionary relationships, we constructed a phylogenetic tree based on the PTS components of the *bgl* operons from six solventogenic *Clostridium* species, together with those from *Escherichia coli* and *Bacillus subtilis*. In addition, the PTS components of the cellobiose, sucrose, and maltose transport systems of *C. acetobutylicum* were included for comparison (Fig. 1A). This analysis revealed that the β-glucoside-specific PTS components of solventogenic Clostridium cluster together with the well-studied homologs from *E. coli* and *B. subtilis*, indicating evolutionary conservation. In contrast, the cellobiose, sucrose, and maltose PTS components of *C. acetobutylicum* were placed in more distant branches, reflecting greater divergence among these sugar transport systems.

The transcriptional activity of *bglT* and *bglH* was examined under various monosaccharides, including glucose, galactose, and fructose, as well as disaccharides like cellobiose, lactose, maltose, and sucrose, using quantitative PCR (qPCR). Our findings revealed that the transcription level of *bglT* and *bglH* was the highest under maltose, followed by cellobiose and sucrose, whereas the transcription was significantly lower under other carbon sources (Fig. 1B). This observation implies that the *bgl* gene cluster is predominantly engaged in the metabolism of disaccharides featuring a glucose-C1 glycosidic bond, whether in the form of an α-glycoside or β-glycoside (e.g., maltose or cellobiose), rather than those with a glucose-C4 glycosidic bond such as lactose. As expected, the Δ*bglT* mutant (Fig. S1) failed to grow on the β-glucosides salicin and arbutin, confirming the essential role of *bglT* in β-glucoside transport. However, the mutant grew as robustly as the wild-type strain on maltose, cellobiose, and sucrose (Fig. 1C), suggesting that the β-glucoside-specific PTS encoded by the *bglGTH* operon is not essential for the metabolism of these specific disaccharides.

### *bglT* is an alternative PTS for the transport of cellobiose and sucrose

**Fig 2:**
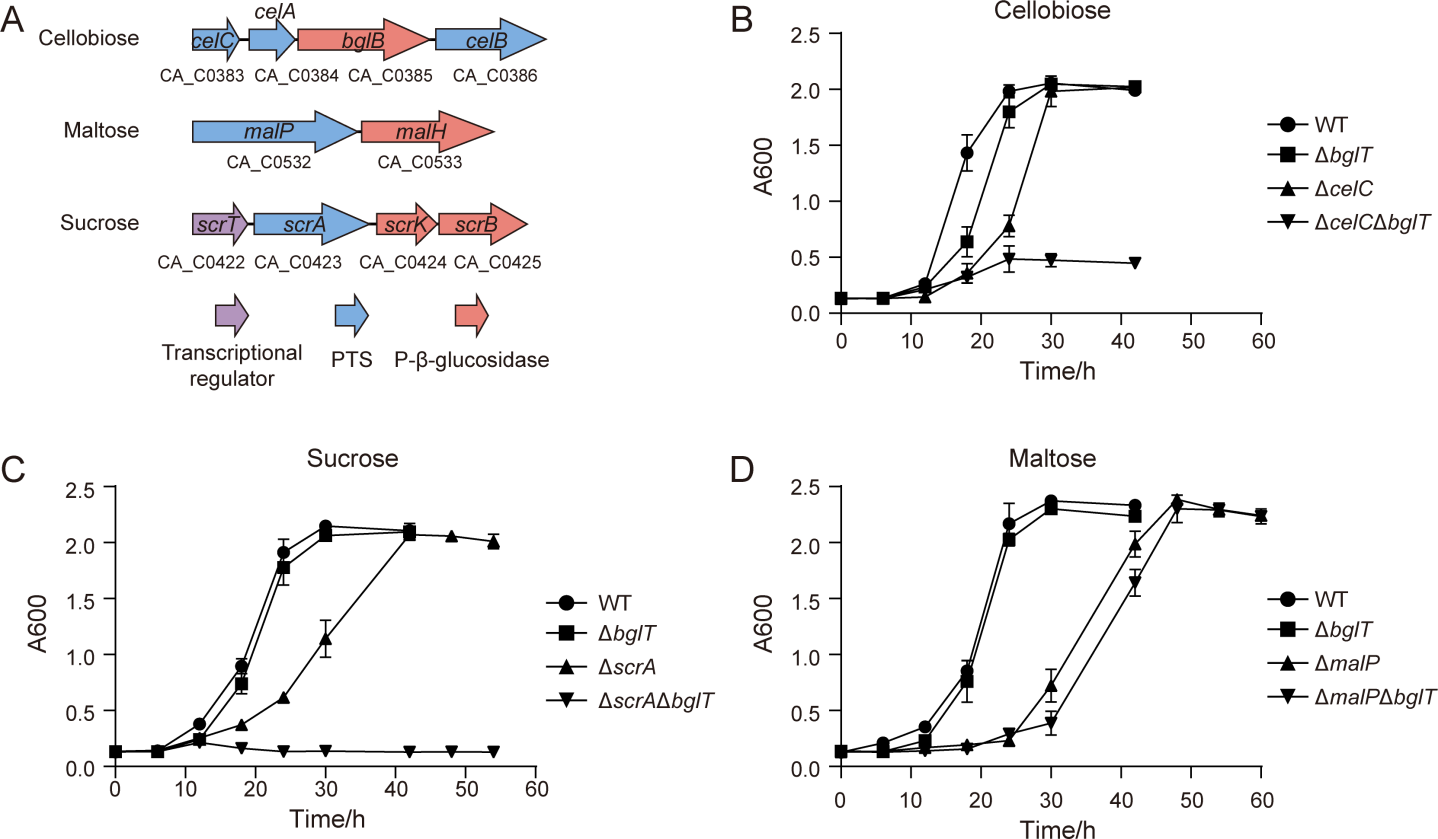
Identification of disaccharide transport systems in *C. acetobutylicum*. (**A**) Genetic organizations of PTSs involved in the transport of cellobiose, maltose, and sucrose. (**B**) Growth curves of *C. acetobutylicum* wild type and mutants (Δ*bglT*, Δ*celC*, and Δ*celC*Δ*bglT*) grown on cellobiose. (**C**) Growth curves of *C. acetobutylicum* wild type and mutants (Δ*bglT*, Δ*scrA*, and Δ*scrA*Δ*bglT*) grown on sucrose. (**D**) Growth curves of *C. acetobutylicum* wild type and mutants (Δ*bglT*, Δ*malP*, and Δ*malP*Δ*bglT*) grown on maltose. Experiments were performed in triplicate, and bars indicate the standard deviation.

To understand how *C. acetobutylicum* prioritizes β-glucoside utilization, we first inspected the three best-characterized PTS operons: CA_C0383–0386 (cellobiose), CA_C0422–0425 (sucrose), and CA_C0532–0533 (maltose). Each showed the canonical IIA–IIB–IIC architecture expected for their substrates (Fig. 2A).

To dissect the contribution of *bglT*, we initially engineered mutants with inactivated PTSs for cellobiose, sucrose, and maltose, named as Δ*celC*, Δ*scrA*, and Δ*malP*, respectively. We then continued to disrupt the *bglT* gene in these individual mutants, yielding double mutants, Δ*celC*Δ*bglT*, Δ*scrA*Δ*bglT*, and Δ*malP*Δ*bglT* (Fig. S1). Subsequently, we investigated the combined effects of the absence of both the specific PTSs and *bglT* on the metabolism of these sugars. Growth curve analysis revealed that the Δ*celC* mutant showed a reduced growth rate, reaching a maximum absorbance at 600 nm (A600) of about 2.0 after a 6 h delay, compared to the wild-type strain under cellobiose. In contrast, the double mutant Δ*celC*Δ*bglT* exhibited minimal growth on cellobiose, with its maximum A600 value only reaching around 0.5 (Fig. 2B). These findings suggest that both the *BglT* and CelABC PTSs contribute to the uptake of cellobiose. While the inactivation of either PTS does not completely impede cellobiose transport, the disruption of the gene encoding celABC results in a markedly slower growth rate.

Similarly, the Δ*scrA* mutant exhibited a slower growth rate under sucrose than the wild-type and Δ*bglT* mutant strains, and the double mutant Δ*celC*Δ*bglT* was unable to grow on sucrose (Fig. 2C). These results indicate that both the BglT and ScrA PTSs are involved in sucrose uptake. Inactivation of either PTS did not entirely prevent sucrose transport, but the disruption of the ScrA system resulted in a markedly slower growth rate. On maltose, both the Δ*malP* mutant and the Δ*malP*Δ*bglT* double mutant exhibited retarded growth relative to the wild-type and Δ*bglT* strains, although all strains ultimately reached a similar final biomass (A600) (Fig. 2D). These results indicate that maltose uptake in *C. acetobutylicum* is primarily mediated by MalP and that additional, MalP-independent transport systems also participate.

In sum, the β-glucoside-specific PTS encoded by the *bglGTH* operon is capable of transporting multiple disaccharides, including cellobiose and sucrose. It serves as an alternate transporter for these sugars, as *C. acetobutylicum* possesses specific transport systems dedicated to each of these sugars.

### The *bgl* gene cluster is transcribed as a single transcription unit

**Fig 3:**
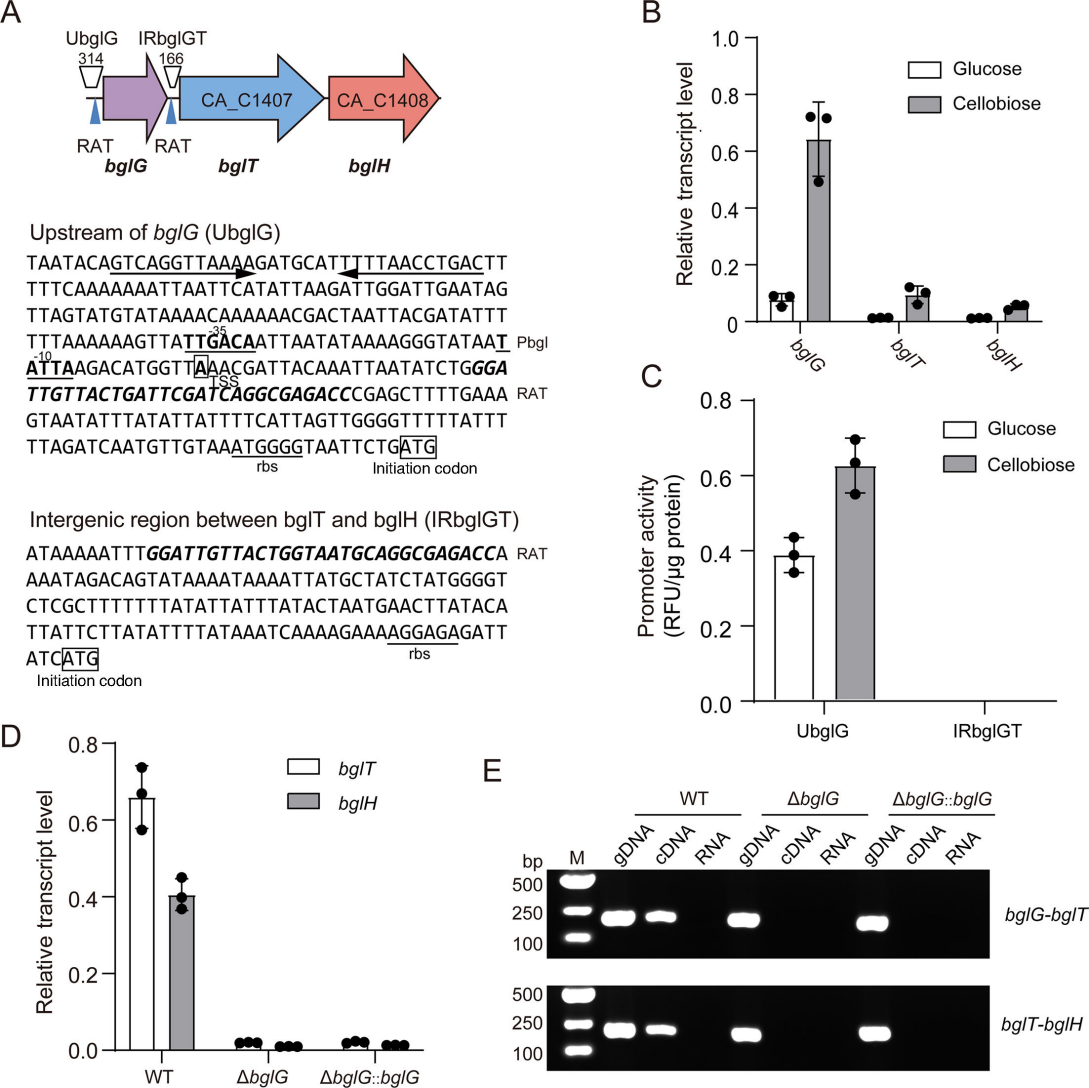
Analysis of the transcription of the *bgl* operon in *C. acetobutylicum*. (**A**) Genetic organization of the *bgl* operon and upstream sequences of *bglG* and *bglT*. Key regulatory elements are annotated. (**B**) Transcription levels of *bglG*, *bglT*, and *bglH* under glucose and cellobiose. Bars represent mean ± SD (*n* = 3), and individual data points are shown. (**C**) Promoter activity of the upstream sequences of *bglG* and *bglT* under glucose and cellobiose. Bars represent mean ± SD (*n* = 3), and individual data points are shown. (**D**) Transcriptional levels of *bglT* and *bglH* in *C. acetobutylicum* wild type, Δ*bglG* mutant, and Δ*bglG::bglG*. Transcription levels were quantified by qPCR using gene-specific primers and normalized to the 16S rRNA gene as an internal reference. Bars represent mean ± SD (*n* = 3), and individual data points are shown. (**E**) Co-transcription analysis of the *bgl* operon. RT-PCR was performed using primers spanning *bglG–bglT* and *bglT–bglH* with cDNA as template, genomic DNA as positive control, and RNA (no reverse transcription) as negative control.

The *bglG* gene, located upstream of *bglT* within the *bgl* gene cluster, encodes a putative *BglG*-type transcriptional regulator that is likely responsible for regulating the transcription of both *bglT* and *bglH*. Consistent with Servinsky et al. (5), we identified two putative RAT elements: one 114–84 nt upstream of the *bglG* start codon and a second 153–124 nt upstream of *bglT*. In addition, a promoter region spanning 273–151 nt was predicted upstream of *bglG* (Fig. 3A). To determine the potential regulatory role of *bglG* in the transcription of *bglT* and *bglH*, we compared the transcription levels of three genes within the *bgl* gene cluster in cultures grown on glucose versus cellobiose using qPCR. The findings revealed that the transcription levels of the *bgl* gene cluster were much higher in the presence of cellobiose than glucose; for example, the transcription level of *bglG* under cellobiose was over six times higher than under glucose, confirming that the transcription of the *bgl* gene cluster was induced by cellobiose. However, the transcription level of *bglG* was more than sixfold higher than that of the other two genes, *bglT* and *bglH*, under both glucose and cellobiose, suggesting the presence of regulatory elements between *bglG* and *bglT* (Fig. 3B).

We evaluated the promoter activity within the intergenic regions of the *bgl* gene cluster using a fluorescent reporter system. Specifically, the 314 bp region immediately upstream of *bglG* (UbglG) was fused to the reporter gene *fbfp*, as was the corresponding intergenic region between *bglG* and *bglT* (IRbglGT). Fluorescence intensity normalized to total protein concentration indicated robust expression driven by UbglG under both glucose and cellobiose conditions, whereas IRbglGT showed no detectable signal (Fig. 3C), ruling out an internal promoter within this intergenic space. To confirm that the entire cluster is transcribed from a single promoter, we employed ClosTron to disrupt the *bglG* gene (Fig. S1), which led to a change in transcriptional polarity. In the resulting Δ*bglG* mutant, transcription of downstream genes *bglT* and *bglH* was abolished and could not be restored by plasmid-based complementation of *bglG* (Fig. 3D). Reverse transcription-PCR (RT-PCR) further revealed loss of the *bglG-bglT* and *bglT-bglH* junction amplicons in the mutant (Fig. 3E). Collectively, these findings suggest that the entire *bgl* gene cluster is transcribed from a single promoter located upstream of the 5 end of *bglG*.

### BglG-RAT switch dictates induction and carbon catabolite repression of the *bgl* operon

**Fig 4:**
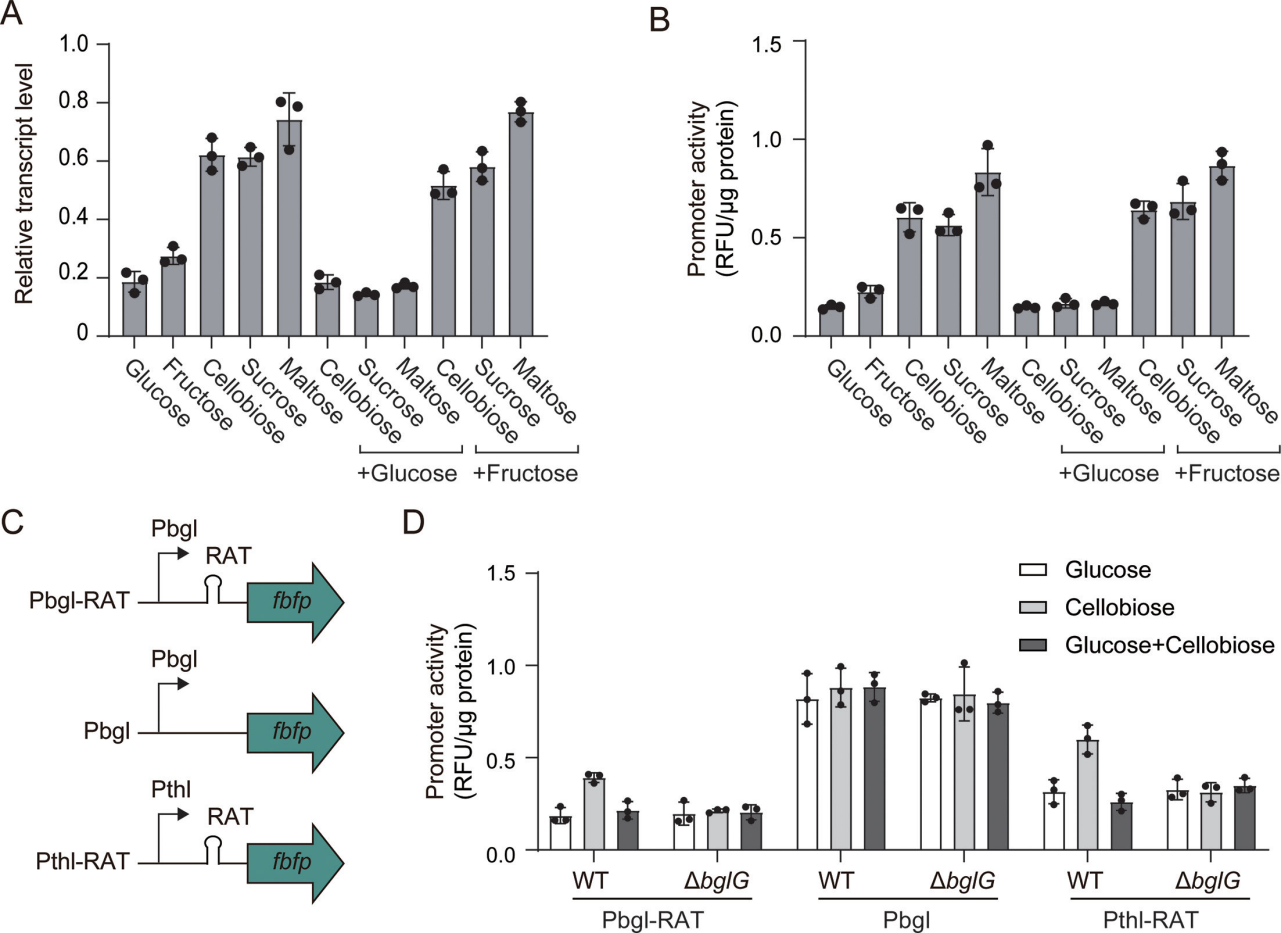
Analysis of transcriptional activity of the *bgl* operon promoter on various carbon sources. (**A**) qPCR quantification of *fbfp* transcripts driven by Pbgl-RAT in wild-type cells grown on glucose, fructose, cellobiose, sucrose, maltose, or mixed sugars. Bars represent mean ± SD (*n* = 3), and individual data points are shown. (**B**) Corresponding promoter activities measured by FbFP fluorescence intensities. Bars represent mean ± SD (*n* = 3), and individual data points are shown. (**C**) Schematic representation of reporter constructs used to separate promoter and RAT contributions: P*bgl*-RAT (native promoter with RAT), P*bgl* (RAT deleted), and P*thl*-RAT (constitutive P*thl* promoter with RAT). (**D**) Promoter activity of the reporter constructs in wild-type and Δ*bglG* strains grown on glucose, cellobiose, or mixed carbon sources. Bars represent mean ± SD (*n* = 3), and individual data points are shown.

To investigate how glucose impacts the transcription of the *bgl* operon, we first fused the entire upstream promoter region (Pbgl-RAT), containing the predicted RAT elements, to the *fbfp* reporter and quantified transcript levels by qPCR. When cultures were grown on cellobiose, sucrose, or maltose as the sole carbon source, *fbfp* transcript levels were markedly higher than on glucose or fructose. Adding glucose to any of the three inducing sugars reduced transcription to the low level observed with glucose alone, whereas fructose co-supplementation had no effect (Fig. 4A). Furthermore, promoter activity measured by FbFP fluorescence closely mirrored these qPCR results. Fluorescence intensities were substantially higher under cellobiose, sucrose, or maltose compared with glucose or fructose and abolished by glucose co-addition (Fig. 4B). Thus, the *bgl* operon is subject to carbon catabolite repression exerted by glucose, but not fructose.

To localize the regulatory determinants, we generated two derivative constructs: (i) P*bgl*, in which the RAT elements were deleted from P*bgl*-RAT and (ii) P*thl*-RAT, in which the native P*bgl* promoter was replaced with the constitutive *Bacillus subtilis* P*thl* promoter (15). These constructs allowed us to separate RAT-mediated regulation from promoter-specific effect (Fig. 4C). Each construct was introduced into both wild-type and Δ*bgl*G strains and assayed under glucose, cellobiose, or mixed carbon sources. The induction of P*bgl*-RAT by cellobiose was completely abolished in the Δ*bgl*G mutant. The constitutive P*thl*-RAT construct exhibited the same dependence on BglG: induction occurred only in the wild type under cellobiose and was lost upon *bglG* disruption. In contrast, when the RAT element was removed (P*bgl*), promoter activity remained uniformly high and identical under all tested carbon sources and in both genetic backgrounds (Fig. 4D). The same regulatory logic applied when sucrose was used as the inducer. RAT-activated expression occurred in the wild type and was abolished by disrupting *bglG* (Fig. S2). Collectively, these results demonstrate that cellobiose and sucrose induction and glucose repression of the *bgl* operon are governed exclusively by BglG and the RAT element, independently of the native promoter context.

### *bglG* enhances transcriptional read-through of the downstream genes of RAT under cellobiose

**Fig 5:**
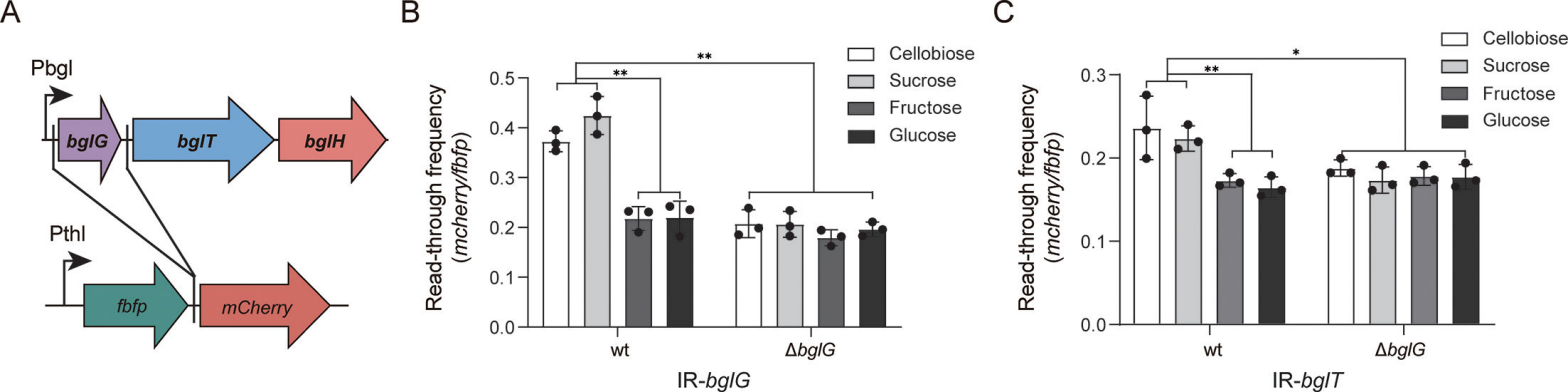
Analysis of the effect of *bglG* on the transcriptional read-through of RAT-adjacent genes. (**A**) Schematic representation of the dual fluorescence reporter system used to study transcriptional read-through. The upstream sequences of *bglG* and *bglT*, containing the RAT site, were inserted between the *fbfp* and *mCherry* genes. (**B and C**) Read-through frequency analysis of the upstream sequences of *bglG* (**B**) and *bglT* (**C**) in *C. acetobutylicum* wild type and Δ*bglG* mutant. The read-through frequency was calculated as the ratio of the transcription level of *mCherry* to that of *fbfp* (*mCherry/fbfp*). Bars represent mean ± SD (*n* = 3), and individual data points are shown. Statistical significance is indicated (*P < 0.05, **P < 0.01; Student’s *t*-test).

To further elucidate the impact of *bglG* on the transcription activity of genes flanking intergenic regions containing RATs, we inserted the RAT-harboring intergenic regions without the promoter sequence (respectively named IR-*bglG* and IR-*bglT*) into a *fbfp-mCherry* bis-cistronic reporter system under the control of the Pthl promoter (Fig. 5A). The relative transcript levels of *fbfp* and *mCherry* from these artificial operons were quantified using qPCR, with the read-through frequency calculated as the ratio of *mCherry* transcription to *fbfp* transcription (*mCherry*/*fbfp*).

In the wild type, the read-through frequency of the IR-*bglG* on the inducing disaccharides cellobiose and sucrose was significantly higher than on glucose or fructose. In the Δ*bglG* mutant, however, read-through was uniformly low and indistinguishable across all sugars (Fig. 5B). Similarly, the read-through frequency of IR-*bglT* was higher on cellobiose and sucrose than on glucose and fructose in the wild type, but no difference was observed between the two sugars in the Δ*bglG* mutant (Fig. 5C). These findings suggest that BglG functions as a sugar-responsive regulator that senses inducing disaccharides such as cellobiose and sucrose, converts RAT structures into antiterminators, and facilitates downstream transcription.

### RAT sequences upstream of *bglG* and *bglT* participate in forming the transcription terminators

**Fig 6:**
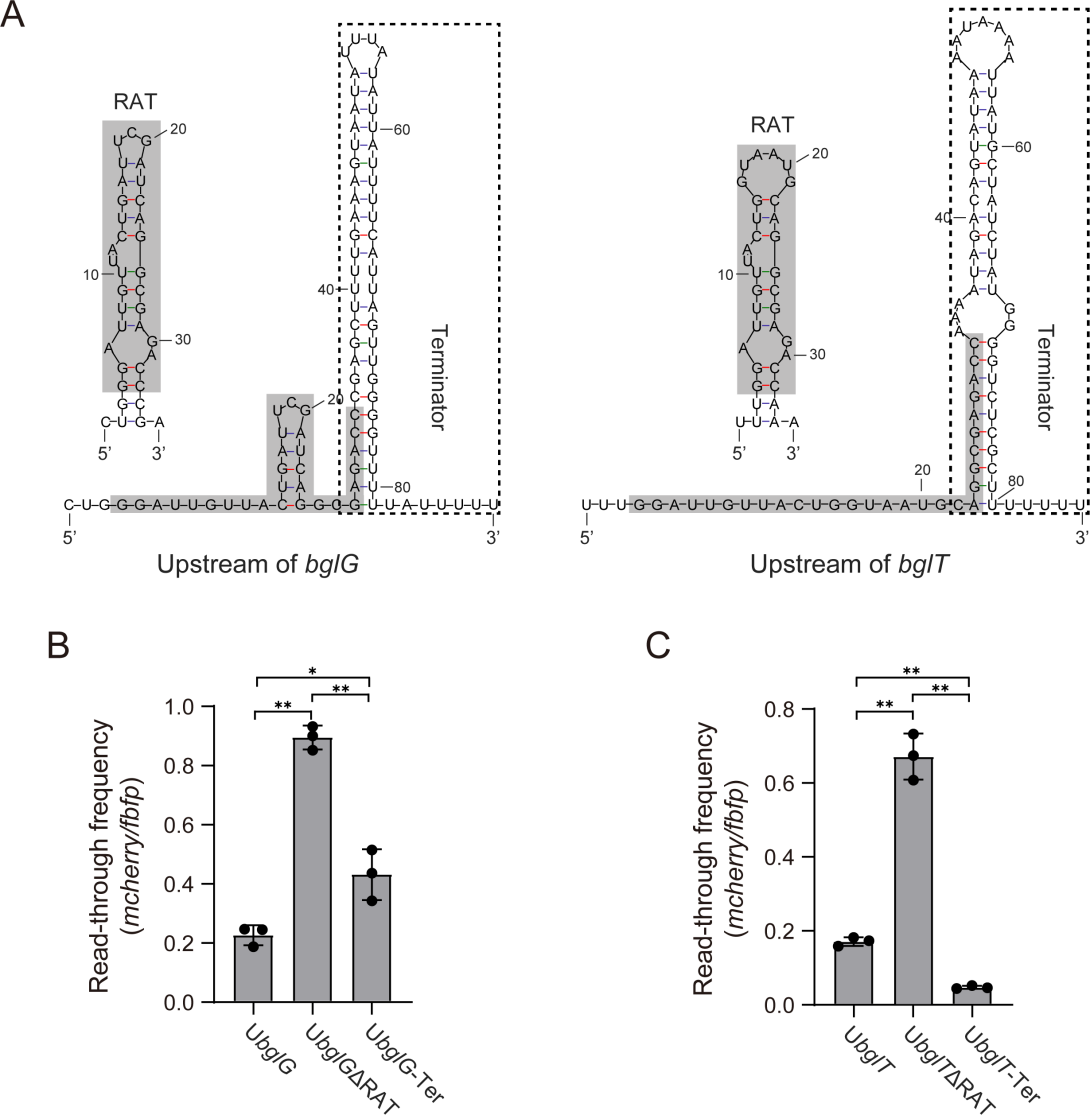
Functional analysis of RAT in the upstream regions of *bglG* and *bglT*. (**A**) Predicted secondary structures of the upstream sequences of *bglG* and *bglT*. The putative RAT antiterminator and terminator structures are highlighted with dark gray boxes and dotted boxes, respectively. (**B**) Read-through frequency analysis of the upstream sequence of *bglG* (U*bglG*) and its RAT-deleted (U*bglG*ΔRAT) and terminator-only (U*bglG*-Ter) mutants in *C. acetobutylicum* wild type. (**C**) Read-through frequency analysis of the upstream sequence of *bglT* (U*bglT*) and its RAT-deleted (U*bglT*ΔRAT) and terminator-only (U*bglT*-Ter) mutants in *C. acetobutylicum* wild type. The read-through frequency was calculated as the ratio of *mCherry* to *fbfp* (*mCherry/fbfp*). Bars represent mean ± SD (*n* = 3), and individual data points are shown. Statistical significance is indicated (*P < 0.05, **P < 0.01; Student’s *t*-test).

The conserved sequence (5′-GGATTGTTACTG-N_6or7_-CAGGCGAGACC-3′) of RAT sequences upstream of *bglG* and *bglT* has the potential to form an imperfect stem-loop structure that functions as an antiterminator. The 3′ end sequences of these RATs, in conjunction with their downstream sequences, form a large stem-loop structure that potentially acts as a terminator. These large stem-loop structures are characterized by a division of their stems into two distinct sub-stems by an intervening unpaired region. Notably, the upper sub-stems are rich in AT base pairs, while the lower sub-stems are GC-rich. Additionally, both large stem-loop structures are adorned with 3′ U-rich tails (Fig. 6A). The architectural features of these large stem loops are indicative of their potential role in transcription termination, functioning as rho-independent terminators.

To confirm the function of the variable stem-loop structures created by RATs, we performed a series of deletions in the RAT sequences upstream of *bglG* and *bglT*. Full deletions were made to generate the mutant sequences U*bglG*ΔRAT and U*bglT*ΔRAT, respectively, while partial deletions aimed at forming the potential terminator structures resulted in U*bglG*-Ter and U*bglT*-Ter. The read-through frequencies of these sequences were subsequently determined using the *fbfp-mCherry* bis-cistronic reporter system under glucose. For comparison, the wild-type sequences from the upstream regions of *bglG* and *bglT* (named U*bglG* and U*bglT*, respectively) served as controls in these experiments. The qPCR results indicated that the read-through frequencies of both U*bglG*ΔRAT and U*bglT*ΔRAT were significantly higher than their respective controls, U*bglG* and U*bglT*, by 85% and 68%, respectively. This suggests that the complete deletion of RATs leads to read-through of their downstream genes due to the involvement of RATs in terminator formation (Fig. 6B and C). Conversely, the read-through frequencies for U*bglG*-Ter and U*bglT*-Ter were lower than those of the RAT-deleted mutants, by 40% and 8%, respectively (*P* < 0.05), indicating that the large stem-loop structures function as terminators. However, the read-through frequency of U*bglG*-Ter is much higher than the control of U*bglG*, suggesting that the RAT of U*bglG*, when part of the terminator structure, enhances the transcription termination together with the downstream large stem loop (Fig. 6B and C).

## DISCUSSION

In this study, we report the identification of a β-glucoside PTS encoded by the *bglGTH* operon in the genome of *C. acetobutylicum*. This operon consists of *bglT* encoding β-glucoside-specific PTS IIBCA component, *bglH* encoding a putative P-β-glucosidase, and *bglG* encoding a putative BglG-type transcriptional regulator. This gene cluster is highly conserved across multiple anaerobic *Clostridium* species. The β-glucoside PTS in *C. acetobutylicum* serves as an alternative PTS responsible for the uptake of cellobiose, sucrose, and maltose. Given that cellobiose is a primary hydrolysis product of cellulose, the most abundant biomass in nature, elucidating its metabolic pathway is crucial for optimizing the fermentation of cellulose-derived substrates (16).

While most PTSs exhibit narrow substrate specificity, certain polysaccharide-specific PTSs can recognize and transport multiple sugars (17). For example, the mannose PTS in *Escherichia coli* can transport mannose, glucose, and N-acetylglucosamine (GlcNAc) (18). Similarly, the GlcNAc PTS in *Clostridium beijerinckii* demonstrates dual substrate specificity for glucose and GlcNAc. This versatility in carbon source utilization is often attributed to the multifunctionality of the EIIC subunits (19), which are responsible for sugar recognition and transport. EIIC subunits involved in the transport of multiple sugars typically possess larger binding sites, enabling them to accommodate structurally similar sugar molecules (20). Servinsky et al. (5) originally identified the cellobiose-inducible *bglGTH* operon in *C. acetobutylicum*. Here, we showed that the BglGTH PTS represents the example of a single system that simultaneously transports two disaccharides, cellobiose and sucrose, as well as the aromatic β-glucosides salicin and arbutin.

The *bgl* operon has been identified in numerous bacterial species and has been extensively characterized in *E. coli* (21–23). In this system, the transcriptional regulator BglG prevents transcription termination by binding to the RAT site, which overlaps with the terminator sequence (24). Previous studies have demonstrated that the phosphorylation state of BglG is the key factor influencing its binding activity (25). In *Bacillus subtilis*, the BglG-type regulator LicT is also controlled by a phosphorylation–dephosphorylation switch mediated by the PTS, in which phosphorylation of specific PRD (PTS regulation domain) regions inhibits RNA binding and antitermination activity (26, 27). In addition, the RAT structure plays a significant role in binding activity. RAT sites exhibit a highly conserved structure, and even minor alterations can affect the recognition specificity of RNA antiterminators (28). Our findings reveal that the *bgl* operon in *C. acetobutylicum* follows a similar regulatory model, with two RAT sites promoting transcriptional read-through (Fig. 5A). We made the novel observation that the transcriptional differences of the *bgl* operon under varying carbon sources are mainly mediated by the RAT site located downstream of the BglG regulatory factor, which connects two intergenic regions.

Regulatory elements based on BglG and RAT have been explored as tools in synthetic biology (29). While existing studies have mainly focused on the RAT itself (28, 30), the terminator structure downstream of RAT exhibits even more variability. Our comparative analysis of the two RAT sites in this study revealed distinct structural features. Similar to previously identified RATs, the two RAT sequences within the *C. acetobutylicum bgl* operon show high conservation, with their secondary structures being notably similar (28). However, this conservation does not fully extend to the downstream terminators. On the one hand, the large neck loops downstream of the two RATs are highly conserved in structure. Compared with classical transcription terminators, the stem loops of these two terminators are significantly longer, both exceeding 20 base pairs, and have low G + C content, indicating that these terminators lack strong stability. These atypical structures are consistent with their functional characteristics, making them susceptible to regulatory factors that may alter their conformation. On the other hand, pairwise sequence alignment revealed that the two terminators share only 56.9% identity and exhibit 26.4% gaps, indicating low similarity, which may account for the different overlap lengths between the RAT and terminator regions. Specifically, U*bglT*-Ter with a longer overlap showed higher termination efficiency compared with U*bglG*-Ter. This difference in termination efficiency partially explains why the expression level of the transcription factor *bglG* under different carbon sources changes less than that of the downstream gene *bglTH*. Therefore, we speculate that a longer overlap between the RAT and downstream terminator sequences is associated with a higher termination efficiency, thereby inhibiting the expression of downstream genes.

In conclusion, this study identified a PTS encoded by a *bgl* operon in *C. acetobutylicum* that participates in the utilization of multiple disaccharides, serving as an alternative transport pathway for cellobiose, sucrose, and maltose. We also elucidate the regulatory mechanism involving the BglG transcriptional regulator and the RAT site. These findings provide a foundation for optimizing the fermentation of cellulose degradation products and enhancing biofuel conversion efficiency in *C. acetobutylicum*. Building on this work, future strategies could integrate engineered cellulase expression with the *bgl* transport system to enable consolidated bioprocessing for direct lignocellulose-to-butanol conversion.

## MATERIALS AND METHODS

### Strains and culture conditions

The bacterial strains and plasmids used in this study are listed in Table S1. *Escherichia coli* strains were cultured aerobically at 37°C in lysogeny broth medium following established protocols (31). *C. acetobutylicum* ATCC 824 was cultured anaerobically at 37°C in clostridial growth medium (CGM), which contains the following components per liter: 0.75 g KH_2_PO_4_, 0.75 g K_2_HPO_4_, 0.71 g MgSO_4_·7H_2_O, 5 g yeast extract, 2 g asparagine, 2 g (NH_4_)_2_SO_4_, 0.01 g MnSO_4_·H_2_O, and 0.01 g FeSO_4_·7H_2_O (32). When required, the media for *E. coli* and *C. acetobutylicum* were supplemented with 100 µg/mL ampicillin, 20 µg/mL tetracycline, or 20 µg/mL erythromycin.

### Plasmid construction

Plasmids designed to disrupt *bglT*, *celC*, *malP*, *scrA*, and *bglG* were constructed based on the control plasmid pSY6. The disrupted targeting sites and intron retargeting primers were designed using an online tool based on the Perutka algorithm (https://clostron.com/) (33). Target regions were sequentially obtained using the IBS/EBSU primer set and splicing by overlap extension PCR (SOE-PCR) with EBS2/EBS1d primers (Table S2) (34). The targeted region was then digested with XhoI and BsrGI and subsequently cloned into the corresponding sites of pSY6 to generate pSY6-bglT, pSY6-celC, pSY6-malP, pSY6-scrA, and pSY6-bglG.

To measure promoter activity, plasmids pMTC6-PbglG, pMTC6-PbglT, pMTC6-PbglGΔRAT, and pMTC6-PthlRAT were constructed based on the shuttle vector pMTC6. The shuttle vector pMTC6 between *E. coli* and *C. acetobutylicum* ATCC 824 contains an oxygen-independent *fbfp* expression cassette under the control of the thiolase (thl) promoter (Pthl) and terminator (15). The PbglG, PbglT, and PbglGΔRAT fragments were amplified by PCR from the *C. acetobutylicum* genome using the primer sets PbglG-F/PbglG-R, PbglT-F/PbglT-R, and PbglGΔRAT-F/PbglGΔRAT-R. The Pthl-RAT fragments were fused by SOE-PCR using the primer set Pthl-RAT-F/Pthl-RAT-R. The PCR products of these fragments were then ligated into pMTC6 to replace the Pthl promoter using the restriction enzymes PstI and NheI.

Plasmids to measure read-through frequency were constructed based on the shuttle vector pMTC9. The *mcherry* gene was inserted downstream of *fbfp* in pMTC6, generating pMTC9, in which both genes are co-transcribed from Pthl (35). The U*bglG* fragment was amplified from the *C. acetobutylicum* genome using PCR with the primer set UbglG_F/UbglG-mCherry_R. The *mCherry* fragment was amplified from pMTC9 using the primer set UbglG-mCherry_F/mCherry_R. The U*bglG* and *mCherry* fragments were then fused by SOE-PCR using the primers UbglG_F/mCherry_R. The resulting PCR product of the fusion fragment was ligated into pMTC9 using the restriction enzymes BglI and EcoRI to replace *mCherry*, resulting in the plasmid pMTC9-U*bglG*. The same method was used to construct pMTC9-UbglGΔRAT, pMTC9-UbglGTer, pMTC9-UbglT, pMTC9-UbglTΔRAT, and pMTC9-UbglTTer.

### Transformation and mutant confirmation

Plasmids were transformed into chemically competent DH5α *E. coli* cells harboring pAN2 for DNA methylation (36). The plasmid vectors were subsequently isolated, and 25 µg of each vector was used for electroporation into *C. acetobutylicum* ATCC 824 as previously described (37). Transformants were selected on CGM plates supplemented with erythromycin. To confirm the presence of intron insertions in the target genes, PCR amplification was performed using gene-specific upstream and downstream primers (Table S2).

### Quantitative reverse transcription-PCR

Total RNA was extracted from mid-log phase cultures of *C. acetobutylicum* using the EZ-10 Total RNA Mini-Prep Kit (Sangon, China). The isolated RNA was reverse transcribed into cDNA using the SPARKscript II RT Plus Kit (Sparkjade, China). Quantitative reverse transcription-PCR was performed on a CFX96 real-time PCR detection system (Bio-Rad, USA) with 2× SYBR Green qPCR Mix (Sparkjade, China). Data were normalized to the 16S ribosomal RNA levels. The primer sequences for qPCR are provided in Table S2.

### Analysis of promoter activity

Potential promoters were predicted using the Softberry BPROM program (http://www.softberry.com/). To evaluate promoter activity, plasmids pMTC6-PBglG, pMTC6-PbglT, pMTC6-PbglGΔRAT, and pMTC6-Pthl-RAT were introduced into *C. acetobutylicum*, and promoter activity was determined by measuring *fbfp*-encoded fluorescence as described previously, with fluorescence values normalized to total protein content. Excitation and emission wavelengths were verified prior to measurement to ensure accuracy (38).

### RNA secondary structure prediction

The Mfold web server (http://unafold.rna.albany.edu/) was used to predict the secondary structures of the regions upstream of *bglG* and *bglT*, using default parameters (39). The resulting stem-loop structures with the lowest free energy (ΔG) were selected for comparison and classification.

## SUPPLEMENTAL MATERIAL

Supplemental material aem.00709-25-s0001.docx Figures S1 and S2; Tables S1 and S2.

ASM does not own the copyrights to Supplemental Material that may be linked to, or accessed through, an article. The authors have granted ASM a non-exclusive, world-wide license to publish the Supplemental Material files. Please contact the corresponding author directly for reuse.
