# Supplementary material for: Identification and regulation of an alternative PTS for disaccharide utilization in Clostridium acetobutylicum
Source: Appl Environ Microbiol. 2025 Oct 8;91(11):e00709-25. doi: 10.1128/aem.00709-25 (PMC12628678; doi:10.1128/aem.00709-25)
Supplement: Supplemental material — Figures S1 and S2; Tables S1 and S2. [file aem.00709-25-s0001.docx]

***Identification and regulation of an alternative PTS for disaccharide utilization in Clostridium acetobutylicum***

Zhenxing Ren^1*^, Zili Qiu^1,2*^, Yali Tian^3^, Mengcheng You^3,4^_,_ Chenggang Xu^2§^

^1^Institute of Applied Chemistry, Shanxi University, Taiyuan 030006, Shanxi Province, China

^2^College of Animal Science and Technology and College of Veterinary Medicine, Zhejiang A&F University, Key Laboratory of Applied Technology on Green-Eco-Healthy Animal Husbandry of Zhejiang Province, Zhejiang Provincial Engineering Research Center for Animal Health Diagnostics & Advanced Technology, Zhejiang International Science and Technology Cooperation Base for Veterinary Medicine and Health Management, China-Australia Joint Laboratory for Animal Health Big Data Analytics, Hangzhou 311300, Zhejiang Province, China

^3^Key Laboratory of Chemical Biology and Molecular Engineering of Ministry of Education, Institute of Biotechnology, Shanxi University, Taiyuan 030006, Shanxi Province, China

^4^Shanxi University of Chinese Medicine, Jinzhong 030619, China

*These authors contributed equally to this work.

^§^To whom correspondence should be addressed. Tel. +86 571 63741392; Email: xucg@zafu.edu.cn

**SUPPLEMENTARY MATERIAL**

**
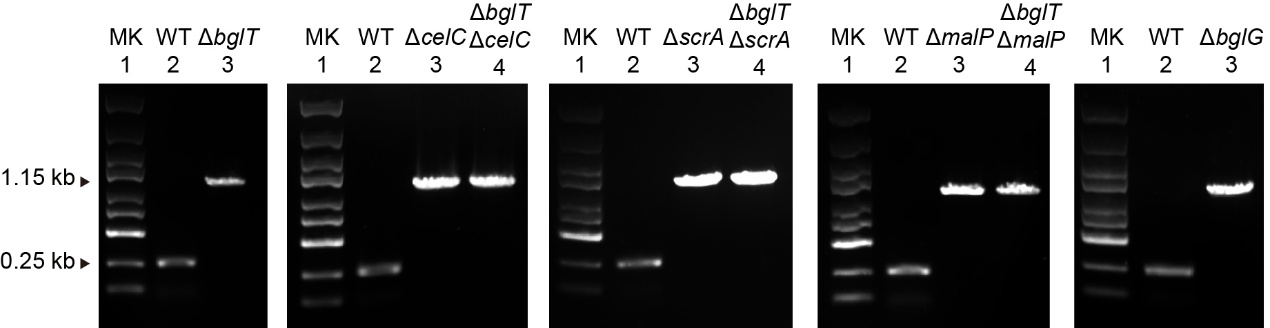
**

**Figure S1. Confirmation of the gene disruption in *C. acetobutylicum* mutants by PCR.**  The *bglT*, *celC*, *scrA*, *malP*, and *bglG* genes were disrupted by inserting an intron. MK, DNA marker; WT, the genome of wild-type *C. acetobutylicum* ATCC824.

**
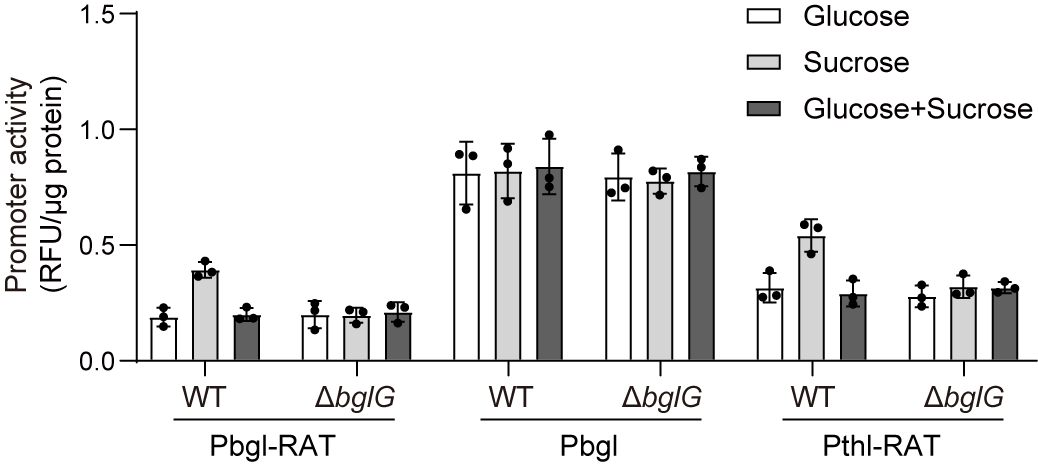
**

**Figure S2. Analysis of *bgl* construct promoter activity.** Promoter activity of the reporter constructs in wild type and Δ*bglG* strains grown on glucose, sucrose, or mixed carbon sources. Bars represent mean ± SD (n = 3), and individual data points are shown.

**Table S1.** Bacterial strains and plasmids used in this study.

| Strain or plasmid | Genotype and/or relevant characteristics | Reference or source |
| --- | --- | --- |
| **Strain** |  |  |
| *Escherichia coli* |  |  |
| DH5α | Host cells for genes cloning and plasmids amplification | Transgene |
| *Clostridium acetobutylicum* |  |  |
| wt | *Clostridium acetobutylicum* ATCC 824 wild-type strain | ATCC |
| Δ*bglT* | Derived from wt, CA_C1407::intron | This study |
| Δ*celC* | Derived from wt, CA_C0383::intron | This study |
| Δ*malP* | Derived from wt, CA_C0532::intron | This study |
| Δ*scrA* | Derived from wt, CA_C0423::intron | This study |
| Δ*bglT*Δ*celC* | Derived fromΔ*bglT*, CA_C0383::intron | This study |
| Δ*bglT*Δ*malP* | Derived fromΔ*bglT*, CA_C0532::intron | This study |
| Δ*bglT*Δ*scrA* | Derived fromΔ*bglT*, CA_C0423::intron | This study |
| Δ*bglG* | Derived from wt, CA_C1406::intron | This study |
| Δ*bglG:: bglG* | Δ*bglG* containing plasmid pMTC6-*bglG* | This study |
| **Plasmids** |  |  |
| pSY6 | MlsR, AmpR, *E. coli*-*C. acetobutylicum* shuttle vector, *ptb* promoter, containing *L.* lactis L1.LtrB intron and *ltrA* | Shao *et al*.(1) |
| pAN2 | *Φ3t I, p15A ori, TetR* | Granted from Zhang *et al*.(2) |
| pMTC6 | MlsR, AmpR, *E. coli- C. acetobutylicum* shuttle vector, containing *fbfp*, thl promoter, thl terminator from *C. acetobutylicum* | Granted from Cui *et al*.(3) |
| pMTC9 | Derived from pMTC6, containing a dual fluorescence reporter system | Wu *et al*.(4) |
| pSY6-bglT | Derived from pSY6, targeting the CA_C1407 | This study |
| pSY6-celC | Derived from pSY6, targeting the CA_C0383 | This study |
| pSY6-malP | Derived from pSY6, targeting the CA_C0532 | This study |
| pSY6-scrA | Derived from pSY6, targeting the CA_C0423 | This study |
| pSY6-bglG | Derived from pSY6, targeting the CA_C1406 | This study |
| pMTC6-bglG | Derived from pMTC6, containing *bglG* from *C. acetobutylicum* instead of *fbfp* | This study |
| pMTC6-PbglG | pMTC6 derivative expression vector harboring promoter sequence of CA_C1406 | This study |
| pMTC6-PbglT | pMTC6 derivative expression vector harboring promoter sequence of CA_C1407 | This study |
| pMTC6-PbglGΔRAT | pMTC6 derivative expression vector harboring promoter sequence of CA_C1406 with the RAT elements deleted | This study |
| pMTC6-PthlRAT | “pMTC6 derivative expression vector harboring the *thl* promoter sequence with the RAT elements deleted.” | This study |
| pMTC9-UbglG | pMTC9 derivative expression vector harboring U*bglG* | This study |
| pMTC9-UbglGΔRAT | pMTC9 derivative expression vector harboring U*bglG*ΔRAT | This study |
| pMTC9-UbglGTer | pMTC9 derivative expression vector harboring U*bglG*Ter | This study |
| pMTC9-UbglT | pMTC9 derivative expression vector harboring U*bglT* | This study |
| pMTC9-UbglTΔRAT | pMTC9 derivative expression vector harboring U*bglT*ΔRAT | This study |
| pMTC9-UbglTTer | pMTC9 derivative expression vector harboring U*bglT*Ter | This study |

**Table S2.** Primers used in this study.

| Primer name | **Sequence (5’→3’)** | **Description** |
| --- | --- | --- |
| bglT-IBS | CCGCTCGAGATAATTATCCTTAAATCACGTTCCAGTGCGCCCAGATAGGGTG | To construct a targeting region for CA_C1407 by SOEing PCR |
| bglT-EBS2 | TGAACGCAAGTTTCTAATTTCGGTTTGATTCCGATAGAGGAAAGTGTCT |  |
| bglT-EBS1d | AGATTGTACAAATGTGGTGATAACAGATAAGTCGTTCCAGATAACTTACCTTTCTTTGT |  |
| EBSU primer | CGAAATTAGAAACTTGCGTTCAGTAAAC |  |
| bglT-F | CACACTGTATTACCCGTTTACG | To validate the mutant of CA_C1407 |
| bglT-R | ACACCTGAAATAGTGTCTAT |  |
| celC-IBS | CCGCTCGAGATAATTATCCTTACAAATCATAATGGTGCGCCCAGATAGGGTG | To construct a targeting region for CA_C0383 by SOEing PCR |
| celC-EBS2 | TGAACGCAAGTTTCTAATTTCGGTTATTTGTCGATAGAGGAAAGTGTCT |  |
| celC-EBS1d | AGATTGTACAAATGTGGTGATAACAGATAAGTCATAATGAATAACTTACCTTTCTTTGT |  |
| celC-F | ATGGATATAGAGCAAATAATAATG | To validate the mutant of CA_C0383 |
| celC-R | TTGTGGTCATTAATTGATCC |  |
| malP-IBS | CCGCTCGAGATAATTATCCTTATAATCCACTACCGTGCGCCCAGATAGGGTG | To construct a targeting region for CA_C0532 by SOEing PCR |
| malP-EBS2 | TGAACGCAAGTTTCTAATTTCGGTTGATTATCGATAGAGGAAAGTGTCT |  |
| malP-EBS1d | AGATTGTACAAATGTGGTGATAACAGATAAGTCACTACCCTTAACTTACCTTTCTTTGT |  |
| malP-F | ACAAATCAAGATATTATGGGTT | To validate the mutant of CA_C0532 |
| malP-R | AGTTAACTCCGAAACTTGAACC |  |
| scrA-IBS | CCGCTCGAGATAATTATCCTTAGGAGCCGCTCTTGTGCGCCCAGATAGGGTG | To construct a targeting region for CA_C0423 by SOEing PCR |
| scrA-EBS2 | TGAACGCAAGTTTCTAATTTCGATTGCTCCTCGATAGAGGAAAGTGTCT |  |
| scrA-EBS1d | AGATTGTACAAATGTGGTGATAACAGATAAGTCGCTCTTGCTAACTTACCTTTCTTTGT |  |
| scrA-F | GTTATAACTGGAATTCATCACAG | To validate the mutant of CA_C0423 |
| scrA-R | GCTTAGTATATCTAAGGTTAAC |  |
| bglG-IBS | CCGCTCGAGATAATTATCCTTAGCAAACCAAGCAGTGCGCCCAGATAGGGTG | To construct a targeting region for CA_C1406 by SOEing PCR |
| bglG-EBS2 | TGAACGCAAGTTTCTAATTTCGATTTTTGCTCGATAGAGGAAAGTGTCT |  |
| bglG-EBS1d | AGATTGTACAAATGTGGTGATAACAGATAAGTCCAAGCAAATAACTTACCTTTCTTTGT |  |
| bglG-F | GCTATAGACTTTATAAAGGATG | To validate the mutant of CA_C1406 |
| bglG-R | CAATTCTATGAAAGAAGAATCT |  |
| GbglG-F | TGTTATTAAATATCTTTTTTATCGTCAT | To amplify the *bglG* |
| GbglG-F | TTAATTTTTCTTTGAGACCCTTT |  |
| bglGT-F | CAAAGGGTCTCAAAGAAAAATTAA | To amplify the *bglG* and *bglT* intergenic region |
| bglGT-R | GGCCAACTTTTCATATTTCAT |  |
| bglTH-F | AGCTACCTGGATGTTATTGA | To amplify the *bglT* and *bglH* intergenic region |
| bglTH-R | ACCTTCACACTGATTAGCCG |  |
| PbglG-F | AACTGCAGTAATACAGTCAGGTTAAAAG | To generate promoter P*bglG* |
| PbglG-R | CTAGCTAGCCAGAATTACCCCATTTACAA |  |
| PbglT-F | AACTGCAGATAAAAATTTGGATTGTTAC | To generate promoter P*bglT* |
| PbglT-R | CTAGCTAGCGATAATCTCTCCTTTTCTTT |  |
| Pthl-F | TGAAATGCGATTAAGCTTGGCTGCAGTATATTGATAAAAATAATAA | To generate promoter P*thl*RAT |
| RAT-R | AGAAGTTTTGCATTTATCATGCTAGCCAGAATTACCCCATTTACAA |  |
| Pthl-RAT-F | TTAGAATGAAAGAAACGCGTAAACGATTACAAATTAATAT |  |
| Pthl-RAT-R | ATATTAATTTGTAATCGTTTACGCGTTTCTTTCATTCTAA |  |
| PbglGdeltaRAT-R | AGAAGTTTTGCATTTATCATGCTAGCAACCATGTCTTAATATTATA | To generate promoter P*bglG* ΔRAT |
| UbglG_F | GAAGATCTCTGGGATTGTTACTGATTCG | To amplify the upstream of *bglG* |
| UbglGΔRAT_F | GAAGATCTCTGCGAGCTTTTGAAAGTAATAT |  |
| UbglG-Ter_F | GAAGATCTCTGGAGACCCGAGCTTTTGAAAGTAATAT |  |
| UbglG-mCherry_F | TAGTTGGGGTTTTTATTTTTATGGTATCAAAAGGAGAAGA | To amplify the DNA fragment of *mCherry* |
| UbglG-mCherry_R | TCTTCTCCTTTTGATACCATAAAAATAAAAACCCCAACTA |  |
| mCherry_R | CGAATTCTTATTTATAAAGTTCATCCA | Downstream primer  To amplify the upstream of *bglT* |
| UbglT_F | GAAGATCTTTTGGATTGTTACTGGTAAT |  |
| UbglTΔRAT_F | GAAGATCTTTTAAAATAGACAGTATAAAATAAAATTATGC |  |
| UbglT-Ter_F | GAAGATCTTTTCAGGCGAGACCAAAATAGACAGTATAAAATAAAATTATGC |  |
| UbglT -mCherry_F | TATGGGGTCTCGCTTTTTTTATGGTATCAAAAGGAGAAGA | To amplify the DNA fragment of *mCherry* |
| UbglT -mCherry_R | TCTTCTCCTTTTGATACCATAAAAAAAGCGAGACCCCATA |  |
| qPCR-bglG -F | TCCGAAAAGTTTAAAATGCTGCT | Intragenic region of CA_C1406, qRT_PCR |
| qPCR-bglG -R | ATTTCCCAAATTAATGGATTGTG |  |
| qPCR-bglT -F | CATTTGCTCAAACAGGCGTAG | Intragenic region of CA_C1407, qRT_PCR |
| qPCR-bglT -R | GAAGCCTGTTATACCTCCACCA |  |
| qPCR-bglH -F | CACGCCTAACTAGTGCCAA | Intragenic region of CA_C1408, qRT_PCR |
| qPCR-bglH -R | ATCAACGGCTCCCAAACCA |  |
| qPCR-fbfp_F | CATGATCAGCCTGGTATAGC | Intragenic region of *fbfp*, qRT_PCR |
| qPCR-fbfp_R | TCTTCTGCAAATACCTGTGCT |  |
| qPCR-mCherry_F | ATTTTCCTTCAGATGGACCTGT | Intragenic region of *mCherry*, qRT_PCR |
| qPCR-mCherry_R | ATTATATGCTCCAGGAAGCTGT |  |

**References**

1. Shao L, Hu S, Yang Y, Gu Y, Chen J, Yang Y, Jiang W, Yang S. 2007. Targeted gene disruption by use of a group II intron (targetron) vector in Clostridium acetobutylicum. Cell Res 17:963-5.

2. Zhang K, Jiang D, Liebl W, Wang M, Gu L, Liu Z, Ehrenreich A. 2023. Confirmation of Glucose Transporters through Targeted Mutagenesis and Transcriptional Analysis in Clostridium acetobutylicum. Fermentation 9:64.

3. Cui GZ, Hong W, Zhang J, Li WL, Feng Y, Liu YJ, Cui Q. 2012. Targeted gene engineering in Clostridium cellulolyticum H10 without methylation. J Microbiol Methods 89:201-8.

4. Wu S, You M, Wang N, Ren Z, Xu C. 2022. Internal Transcription Terminators Control Stoichiometry of ABC Transporters in Cellulolytic Clostridia. Microbiol Spectr 10:e0165621.
